# The Columbus Meeting of the Georgia Medical Association

**Published:** 1903-05

**Authors:** 


### THE COLUMBUS MEETING OF THE GEORGIA MEDICAL ASSOCIATION.

THE Georgia Medical Association held its annual meeting at Columbus, April 15, 16, 17. The attendance was unusually large and the local members of the profession displayed great zeal in the entertainment of their guests. A banquet and a Bohemian smoker were tendered the visiting members, and a boat-ride on the river, all of these diversions being very much enjoyed. The list of papers was large and of scientific value. A paper was presented by Dr. H. McHatton of Macon on the servant question. The paper, together with one of similar character by Dr. E. C. Thrash read last year, was ordered printed at the expense of the Association for distribution among the members of the legislature as bearing on the establishment of a State Board of Health. To the furtherance of this object a committee was appointed under the chairmanship of Dr. Chas. Hicks of Dublin to confer with a similar committee from the lower house to endeavor to secure the passage of the State Board of Health bill. The program committee was abolished and this feature included among the duties of the secretary-treasurer. There were eighty-two new members enrolled.

The Association, after electing officers, adjourned to meet at Macon, April, 1904. The officers for the ensuing year were elected as follows: President, H. McHatton, Macon; Vice-President, J. H. McDuffie, Columbus; 2d Vice-President, E. C. Thrash, Meriwether; Alex. Mack, Dublin, to succeed F. W. McRae on Board of Censors.

